# Serum lipid profile of Parkinson's disease patients: A study from the Northwest of Iran

**DOI:** 10.22088/cjim.12.2.155

**Published:** 2021-03

**Authors:** Samira Saedi, Mohsen Hemmati-Dinarvand, Haleh Barmaki, Zohreh Mokhtari, Hadis Musavi, Mohamad Valilo, Ali Mota, Soleiman Mahjoub

**Affiliations:** 1 Department of Medicine Microbiology, Faculty of Medicine, Kurdistan University of Medical Sciences, Sanandaj, Iran; 2 Department of Clinical Biochemistry and Laboratory Medicine, Faculty of Medicine, Shiraz University of Medical Sciences, Shiraz, Iran; 3 Department of Laboratory Medicine, Faculty of Paramedical Sciences, Shahid Beheshti University of Medical Sciences, Tehran, Iran; 4 Department of Clinical Biochemistry and Laboratory Medicine, Faculty of Medicine, International Branch, Shahid Beheshti University of Medical Sciences, Tehran, Iran; 5 Student Research Committee, Babol University of Medical Sciences, Babol Iran; 6 Department of Clinical Biochemistry, School of Medicine, Babol University of Medical Sciences, Babol, Iran; 7 Department of Clinical Biochemistry and Laboratory Medicine, Faculty of Medicine, Tabriz University of Medical Sciences, Tabriz, Iran; 8 Cellular and Molecular Biology Research Center, Health Research Institute, Babol University of Medical Sciences, Babol, Iran; & Samira Saedi and Mohsen Hemmati-Dinarvand contributed equally in this article

**Keywords:** Triglycerides, LDL, HDL, Cholesterol, Parkinson Disease

## Abstract

**Background:**

Parkinson's disease (PD) is defined as a long-lasting, neurological illness. Low levels of serum lipid fractions are related with a high risk of PD. Current investigation was designed to evaluate the concentration blood lipid fractions in patients suffering from PD and compared with healthy subjects.

**Methods:**

This case-control study was conducted from February 2016 to September 2018 in Tabriz University of Medical Sciences, Tabriz, Iran. The present investigation consisted of 75 persons who had PD and 75 normal people. The blood levels of lipid fractions were measured by concentrations of total cholesterol (TC), serum triglycerides (TG), low-density lipoprotein (LDL-C), high-density lipoprotein (HDL-C), and total cholesterol. The results were analyzed with SPSS software using Kolmogorov-Smirnov, chi-square, and student's t-test.

**Results:**

Serum level of TG was remarkably lower in patients with PD (111.92±8.75 mg/dL) compared with healthy subjects (123.64±9.97 mg/dL, P=0.008). Furthermore, we saw an important difference in the level of LDL-C (P=0.001) and TC (P=0.004) between the two groups. However, there was not any observed meaningful difference in the serum concentrations of HDL-C between the studied groups (P=0.135).

**Conclusion:**

Our results showed that the serum concentration of TG, LDL-C, and TC are noticeably lower in the PD suffering patients. Further investigations are needed to provide comprehensive information on the participants' cognitive layout and subsequent actions.

Parkinson's disease (PD) defined as a long-lasting and neurological illness occurs in people over 65 years old, in PD patients, part of the disease process develops as cells are destroyed in certain parts of the brain stem in the substantia nigra area that produces dopamine normally followed by stimulation of cell death during the development of the illness (1-3). The frequency of lipid fractions and total cholesterol (TC) metabolism occur in the pathogenesis and progression of PD. TC has an important function in the normal functioning of the neuronal cell membranes and synapses, and also plays a prominent role in maintaining their function and structure (4). It has been shown that there is a relationship between lipid fractions and the structure and localization of the α-synuclein, as the main component of the Lewy body pathology that is present in the brain tissues of the individual who is suffering from PD (5, 6). Researchers found that blood levels of TC were lower in subjects who had PD compared to healthy subjects (7, 8). Furthermore, the serum TC concentration has a main function in determining the serum levels of coenzyme Q10, which is a good antioxidant and mitochondrial electron receiver.

As shown previously, coenzyme Q10 plays an effective role in animal model studies and clinical studies on PD (9, 10). A previous study showed a high concentration of LDL-C with low risk of PD. Thus, there is a reverse relationship between the levels of lipid fractions and the risk of PD (11). Moreover, it is suggested that the serum level of HDL-C has a positive relationship with duration of PD. Therefore, the concentration of lipid fractions is associated with the development and duration of PD (12). However, another investigation reported contradictory findings (13). De Lau *et al*. reported that high HDL-C level in middle-aged persons is associated with cognitive disorders in the elderly, and it is a prospective risk factor for PD occurrence (14). 

In addition, a previous study exhibited that the concentration of the serum cholesterol and TG in subjects who had PD were meaningfully lower than the normal people (15). Scigliano *et al*. showed that TG was meaningfully lower in individuals suffering from PD compared to the control groups (15). Conversely, an investigation on lipid profiles of PD patients did not approve the relationship between TC levels and progress of PD (16).

This study evaluated the serum concentrations of TC, TG, HDL-C, and LDL-C in patients with PD and the healthy group. The possible relationship between the concentration of lipid fractions and risk of PD was investigated previously, but their results were inconsistent. We conducted this study to assess the lipid profile concentration among patients with PD and control group. We assumed that several factors such as race, nutrition, and lifestyle affect clinical factors; therefore, it is likely that our results will be different from those previously obtained in other regions.

## Methods

**Patients and study design:** The present investigation is a case-control study that included 75 Parkinson's patients and 75 control subjects. The case group included all patients with PD who were referred to Imam Reza Hospital, Tabriz, Iran. Also, the control group subjects were selected randomly between healthy people that were best matched with the case group. Based on the United Kingdom PD Society Brain Bank of Clinical Diagnostic Criteria (17), subjects who were suffering from PD were selected from the Imam Reza Hospital, Tabriz, University of Medical Sciences, Tabriz, Iran. These patients were evaluated by a neurologist and their disease was confirmed on the basis of clinical symptoms. All those who suffer from PD were recruited at stages < 3 based (they were capable to stand and walk autonomously) using Hoeh and Yahr (H & Y) assortment (18). This study was confirmed by the Ethics Committee of Clinical Research in Tabriz University of Medical Sciences, Iran (Ethical code: IR.TBZMED.REC.1395.823). Every subject in this investigation received enough description about the research and informed consent. This study was conducted from February 2016 to September 2018. Patients who had gout, multiple sclerosis, secondary Parkinsonism, migraine, epilepsy, hepatic disease, chronic inflammation, metabolic syndrome, and hematologic diseases were excluded. Demographic features were obtained at the sampling time. The age range of all the participants was between 59 to 72 years old. 

**Sample preparation and measures: **Phlebotomy (5 mL) at early morning was performed on the samples obtained from peripheral vein of the participants after 12 h fasting. After the clot was formed, the serum was separated through centrifugation 3000 rpm at Lab temperature. To assess the lipid fractions, the serum was moved into micro-tubes and saved at −70°C. The serum concentrations of lipid fractions were measured by levels of TC, LDL-C, HDL-C and TG along with comparison of their means at the Clinical Laboratory of Rad in Tabriz, Iran. Briefly, the serum levels of TC, TG and HDL-C were measured by Hitachi Clinical Autoanalyzer 7600 (Hitachi, Tokyo, Japan). LDL-C was calculated using Friedewald formula as follows: LDL-C (mg/dl) = TC (mg/dl) – HDL-C (mg/ dl) – 0.2×TG (mg/dl) (19).

**Statistical Analysis: **The results were analyzed by SPSS software (Version 16), and Graph Pad Prism software (Version 6.07). Kolmogorov-Smirnov test was applied to evaluate the normality of quantitative variables. Quantitative and qualitative variables were mean±SD and frequency (percentage), respectively chi-square and independent sample t-test were used to analyze the data. A p<0.05 was considered as significant. 

## Results

A total of 150 samples were taken from 75 individuals with PD and 75 control subjects in this study. Adjustment of certain factors such as age, gender, hypertension, diabetes mellitus, BMI, and smoking was done between the two groups, so we did not see a meaningful difference (table 1).

**Figure 1:**
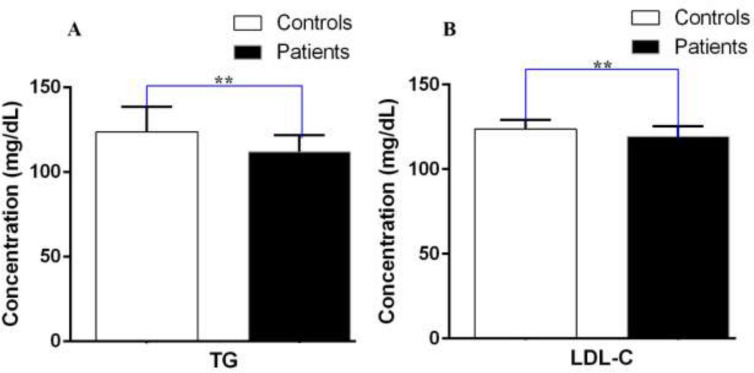
Equivalence of the serum levels of (A) TG, (B) LDL-C. **p-value < 0.05

**Figure 2:**
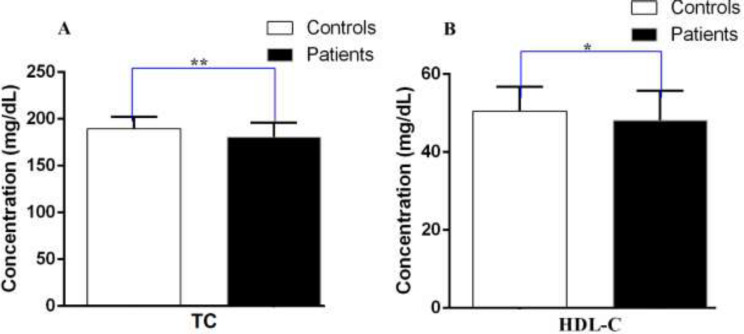
Equivalence of the serum levels of (A) TC, (B) HDL-C

The mean levels of TG (111.92±8.75 vs. 121.64±9.97, P=0.008) and LDL-C (119.18±6.13 vs. 123.67±5.44, P=0.001) were remarkably lower in persons who had PD compared to the healthy subjects (Fig. 1 A and B). In addition, TC (180.33±7.40 vs. 189.49±12.36, P= 0.004) in subjects suffering from PD was meaningfully lower than healthy subjects (fig. 2 A). Serum HDL-C levels in PD suffering subjects and control group (48.06±4.62 vs. 50.42±6.27, p=0.135) were not significantly different (fig. 2B). We also investigated the relationship between the serum levels of TG, LDL-C, HDL-C, and TC with age, gender and disease duration in PD suffering individuals. In certain quantities, the variables in the subgroups increased slightly, but the relationships were not significant (table 2). 

**Table 1:** Demographic results of the subjects suffering from PD and control subjects

**Variables**	**PD patients (n=75)**	**Healthy group (n=75)**	**p-values**
Gender			-
Male (n)	46	50	
Female (n)	29	25	
Age (years)	65.70±6.32	64.35±3.75	0.249
Disease duration (years)	5.91±1.35	0	-
Hypertension (n)	16	13	0.634
Smoking (n)	9	7	0.329
Diabetes mellitus (n)	5	3	0.456
BMI (kg/m²)	26.39±1.65	25.98±1.26	0.211

p-value was reported based on Independent Sample t-test.

p-value was reported based on Chi-Square test. PD = Parkinson disease; BMI = Body mass index. Values are as Mean±SD

Analysis of the data was done using Student's t-test. TG = triglyceride; LDL-C = low-density lipoprotein.

*p-value = not significant. **p-value < 0.05. Analysis of the data statistical was done by independent sample t-test. TC = total cholesterol; HDL- C = high-density lipoprotein.

**Table 2:** Subgroup assessment of serum TG, LDL-C, TC and HDL-C in PD patients

**Variable (N)**	**TG**	**LDL-C**	**TC**	**HDL-C**
Gender				
Male(46)	118.23±5.8	125.34±5.2	185.16±9.4	45.75±6.4
Female(29)	112.61±7.4	119.49±4.7	182.53±7.5	49.28±7.1
^*^p- value	0.450	0.585	0.347	0.164
Age				
≤65 (26)	115.14±6.9	121.79±8.4	183.11±7.4	48.53±4.8
˃65 (49)	122.84±7.2	127.41±6.5	181.65±5.8	46.86±5.7
^*^p- value	0.065	0.795	0.710	0.768
Disease duration				
≤3 years (23)	112.46±7.8	120.64±5.9	185.75±6.3	49.76±7.4
> 3 years (52)	114.65±6.1	122.17±8.1	180.94±7.5	48.11±6.5
^*^p- value	0.882	0.786	0.475	0.863

PD = Parkinson disease;  p-value was reported based on Student's t-test.

TG=Triglycerides; LDL-C=Low-density lipoprotein; TC=Total cholesterol; HDL-C=High-density lipoprotein. Values are as Mean±SD

## Discussion

Low serum concentrations of TC and LDL-C can be related to a high prevalence of PD (20). This study evaluated the blood concentrations of TC, HDL-C, LDL-C, and TG in patients who are suffering from PD. The present results showed that the concentrations of serum TG, LDL-C, and TC were meaningfully lower in subjects with PD compared to control individuals. These findings were in line with previous studies; case-control studies on patients with PD showed that the blood levels of TC, LDL- C, and TG were markedly lower in individual with PD compared to healthy group. This claim suggests that lower lipid fractions may be related to an enhanced risk of PD (15, 19, 21-23). Following this aim, in the large population-based AMORIS’ study counting about 600 000 contributors with a follow-up of >20 years, F. Fang et al. found a statistically significant relationship between a higher level of TC, LDL-C, and TG with a lower risk of PD (24). X. Huang et al. in a study on Japanese–American men with PD, reported that lower serum level of LDL-C is related with enhanced PD risk (11). Interestingly, an investigation conducted by Rotterdam cohort on women showed a relationship between a lower TC and an increased risk of PD (14). X. Guo et al. stated that the lower concentration of lipid fractions in patients with PD can be previous to the PD detection since the premorbid status of lower lipid fractions increases the risk of PD (20). X. Huang *et al.* offered the first initial evidence that the lower serum concentration of TC may be associated with the relatively faster development of PD symptoms (25). 

In addition, several prospective investigations reported that high concentration of TC is related with low PD risk (14, 22). X. Guo et al. believed that a decrease in the food intake by PD patients leads to lower lipid profiles in these patients (20). However, Hu. G *et al.* suggest that high level of TC at baseline is related with an enhanced risk of PD. (13). Moreover, it was shown that an increase of skin-fold thickness to triceps assessed in middle-age was related to an enhanced risk of PD (26). Furthermore, enhanced BMI was related with an elevated risk of PD autonomous of other risk factors (27), in addition, central obesity is a reason for enhancing the risk of PD among nonsmokers (28). 

Though most investigations, containing prospective cohort studies described a reliable inverse relationship of TC, LDL-C, and TG with PD, the relationship might still be caused by residual confounding or converse causation (24), For instance, a former investigation stated a shared genetic etiology between TC/TG and PD, suggesting that genetic confounding could play a role to the following associations of serum concentrations of TC/TG with PD risk. Moreover, altered lipid concentration might represent a prodromal sign of PD, which might debut long formerly motor indications and remains as an anxiety even for prospective cohort investigations with long follow-up (29). Previously, it was shown that cholesterol also plays a role in a series of vital biological activities such as cell repair or degeneration as a neuro-steroid precursor (30-32). Kaikkonen *et al*. stated that some of the factors might decrease the frequency of PD in strategies that use a high concentration of lipid fractions. High serum cholesterol decreases PD through increasing the protection of neurons effect of coenzyme Q10 because blood TC is the main factor of coenzyme Q10 in the blood (33). Earlier research on PD animal model showed that coenzyme Q10 had protection effects versus the dopamine degeneration, absence of tyrosine hydroxylase neurons and the stimulation of *α*- synuclein inclusions in the substantia nigra by diminishing oxidative stress and mitochondria dysfunction (34, 35). Furthermore, a cholesterol precursor, lanosterol, was found to be significantly lower in the nigrostriatal region of a PD animal model, revealing altered lanosterol metabolism during PD pathogenesis, whereas exogenous addition of lanosterol was found to resurrect dopaminergic neurons from death (36). Furthermore, cholesterol might also modulate the progress of PD by means of other ways. For instance, iron deposit and deposit (29) have been associated in the pathology and pathogenesis of PD and substantia nigra might be the primary region for iron accumulation (36 from ref). Cholesterol can bind ferrous iron in complexes by neuromelanin, which are later deposited in lysosomes through autophagy, preventing therefore iron-induced oxidative stress and neurodegeneration (24).

In the present study, there was also little difference in HDL-C between individuals who had PD and control group, but it was not statically considerable, which is consistent with the previous study. (14, 37-39). However, X. Huang *et al*. exhibited the high serum concentration of HDL-C in females which is accompanied by a lower prevalence of PD (40). Discrepancy in the serum concentration of hormones between genders (male and female) might be involved in such gender discrepancy (20). Our results in the subgroup analysis of the serum concentrations of TG, LDL-C, TC and HDL-C in subjects suffering from PD showed that there was no statistically important difference in age, gender, duration of illness and the measured variables on the serum level of PD patients. However, our results showed that TG, LDL-C, and TC in men were higher than women; also, HDL-C levels in women were higher than men. Furthermore, the serum level of TG and LDL-C in PD patients aged > 65 years old was higher than that ≤ 65 years old. Also, TC and HDL-C in PD patient age > 65 years old were lower than those aged ≤ 65 years old. 

While lipid fractions may have an effect on the etiology of PD or clinical symptoms, it is also believed that lower lipid fractions can be regarded as common markers for the advanced risk of PD. Therefore, comprehending the principal mechanism of the relationships between lower lipid fractions and an increased risk of PD may have an important effect in understanding the main aspects of PD prevalence. 

Our study had some limitations; first, we measured the lipid profiles in patients with PD after diagnosis of their disease, while monitoring the lipid profile status in the high risk subjects may better reveal the relationship between serum lipid profiles and the incidence of PD. Second, we could not control some aspects that may influence the risk of PD, such as smoking, and caffeine consumption. The third limitation was the relatively small sample size and lack of aftermath results from the subject who had PD.

To conclude, the present results exhibited that serum concentration of TC, LDL-C, and TG in subjects with PD were lower than the healthy individuals. Lipid abnormalities appear to be associated with PD, as further studies can investigate the underlying mechanism in this area, which is more dependent on diet and lifestyle.
